# What is known about pathways to mental health care for Australian Aboriginal young people?: a narrative review

**DOI:** 10.1186/s12939-018-0727-y

**Published:** 2018-01-27

**Authors:** Alexandra Kilian, Anna Williamson

**Affiliations:** 1 McMaster Health Forum, 1280 Main Street West, MML-417, Hamilton, ON L8S 4L6 Canada; 2 Centre for Informing Policy in Health with Evidence Research, Sax Institute, Level 13, Building 10, 235 Jones St, Ultimo, NSW 2007 Australia

**Keywords:** Mental health, Children, Youth, Adolescents, Aboriginal, Indigenous, General practice, Primary care

## Abstract

**Objectives:**

To (1) gain an understanding of current trajectories of Aboriginal young people through the mental health care system in Australia; (2) summarize what mental health care pathways have been developed or evaluated to guide mental health care delivery for Aboriginal young people; and (3) identify barriers and facilitators to the adoption of effective mental health care pathways for Aboriginal young people.

**Methods:**

Databases, including, AMED, Embase, Global Health, Health and Psychosocial Instruments, Healthstar, MEDLINE, PsychINFO via Ovid, CINAHL via EBSCO, The Cochrane Library, Indigenous Collections, Informit and Health Systems Evidence, were searched to identify evidence concerning mental health service delivery for Aboriginal young people in a primary care setting.

**Results:**

We did not identify any reports or publications explicitly describing the current trajectories of Aboriginal young people through the mental health care system in Australia. Furthermore, we were unable to locate any mental health-related treatment pathways which had been explicitly developed or modified to meet the needs of Aboriginal young people. The use of appropriate assessment tools, engagement of family and community, flexibility, and central coordination have been identified in the literature as potential facilitators of culturally appropriate mental health service delivery for Aboriginal children and adolescents.

**Conclusions:**

Aboriginal children and adolescents may face additional difficulties navigating the mental health care system in Australia due to complex socio-cultural factors and the dearth of culturally appropriate and effective mental-health related treatment pathways. Additional research regarding (1) practice trends in Aboriginal settings and (2) how Aboriginal child and adolescent mental health can be improved is urgently needed to inform clinical practice and improve mental health service access and outcomes for Aboriginal young people in Australia.

## Background

The few studies which have sought to estimate the burden of mental health disorders among Aboriginal young people in Australia have found it to be significantly greater than amongst Australian young people in general [1–4]. For example, two major studies involving 4–17 year-old Aboriginal children reported prevalences of high risk of emotional or behavioural problems of 28% [2] and 24% [3] respectively. Both of these studies showed that the prevalence of Aboriginal children at high risk for social and emotional difficulties differed somewhat by age, with slightly lower prevalences seen among adolescents than younger children. Comparatively, only 7.6% of all children aged 4–15 years in New South Wales have been estimated to be at high risk [5]. A robust, systemic response to this discrepancy is needed, given the potential acute and long term consequences of poor childhood mental health on social, academic, developmental, and health outcomes [6–8].

The elevated mental health burden observed amongst Aboriginal children is related to Australia’s history of colonization which has had profound intergenerational impacts on the social and emotional wellbeing (SEWB) of Australian Aboriginal people and has led to ongoing social, political, and economic marginalization [9]. Additionally, multiple sources have suggested that many current services and care settings are insufficiently culturally safe for Aboriginal people and that mainstream protocols have not been adequately modified to respond to the needs of this population [10–14]. The urgent need to improve the mental health system's response to the needs of Aboriginal young people is starkly illustrated by the high rates of self-harm and suicide noted within this group [15, 16].

Goldberg and Huxley’s model of pathways to care provides a framework for understanding the movement of patients into and through the mental health-care system [17]. The model proposes that the system can be conceptualized in terms of five discrete sectors of care, from community to inpatient specialist mental health care. Sectors are separated by filters that reflect factors such as accessibility and patient values, as well as decisions related to consultation, diagnosis, referral, and admission processes [17, 18]. Care pathways have the potential to improve inter-professional collaboration, with potential positive impacts on patient outcomes [19–25]. Research suggests that service providers in Aboriginal communities value defined, written protocols for the screening and assessment of mental health challenges [12]. Such care pathways have been established in Australia in regards to a range of child and adolescent mental health problems such as depression [26], anxiety [27], and ADHD [28]. However, it is not known if or how well these work for Aboriginal young people.

Almost all of the small amount of research to date which has examined Aboriginal child and adolescent mental health has focused on general social and emotional wellbeing. Thus, almost nothing is known about the prevalence or correlates of specific mental disorders amongst Aboriginal young people. In keeping with this, this review examines ‘mental health’ in a broad sense, defining ‘mental health problems’ as “behavioural or emotional problems or associated impairment” [29]. While the evidence base does not currently exist to allow comment on specific disorders separately, we acknowledge that this broad term includes disorders such as anxiety, depression, and attention deficit hyperactivity disorder, as well as the psychotic disorders. ‘Social and Emotional Wellbeing’ refers to a holistic understanding of health which includes connections of the individual to culture and community, and has been described in detail elsewhere [11, 30]. Though they are distinct concepts culturally and in practice, mental health and social and emotional wellbeing will be used interchangeably in this review, as has been the case in the research literature on Aboriginal child and adolescent mental health to date. The term ‘young people’ will refer to all individuals of 0–18 years, inclusive. The term Aboriginal will refer to the Aboriginal and Torres Strait Islander population of Australia.

This narrative review explores what is known about ideal care pathways in mental health, with a specific focus on (1) Aboriginal young people in Australia, and (2) the Australian general practice (GP) setting. This review focuses on the GP setting as, in Australia, general practitioners (GPs) act as a gateway to specialist health services, including mental health services [31]. A better understanding of current trends, and barriers and facilitators to mental health care for Aboriginal children and adolescents in this setting has the potential to contribute to systemic improvements in an area of great need.

The primary questions that guided the research process were as follows: (1) What are the current trajectories by which Aboriginal young people traverse the mental health care system; (2) What pathways to mental health care have been developed or evaluated to guide primary mental health care delivery for Aboriginal young people; (3) What barriers and facilitators exist in the adoption of effective mental health care pathways for Aboriginal young people; and (4) What recommendations have been made for improvements to mental-health service delivery for Aboriginal young people?

## Methodology

### Literature search

From May 2016 to July 2016, a comprehensive iterative search of databases was conducted to find published guidelines, as well as descriptive studies, reports, and reviews that concern the delivery of mental health care for Aboriginal and Torres Strait Islander young people in a primary care setting. A wide range of databases were searched, including: AMED, Embase, Global Health, Health and Psychosocial Instruments, Healthstar, MEDLINE, PsychINFO via Ovid, CINAHL via EBSCO, The Cochrane Library, Australian Indigenous Health*Info*Net and Health Systems Evidence. The search was filtered by ‘*Humans/English language/child or adolescent (0-18)/abstract available’* where possible. No limits were set for date of publication. Additional references were identified via the ‘snowballing’ method, wherein reference lists of reviewed publications were assessed. This process was supplemented by searching Google Scholar. The initial search algorithm was as follows: [(Aboriginal/Indigenous/“Torres Strait Islander”) AND (“mental health”/“mental illness”/“Social and emotional wellbeing”/“psych$) AND (“young people OR child$ OR adolescent$] AND “(trend$ OR review$ OR pathway$ OR methodolog$ OR protocol$ OR guideline$ OR algorithm$) AND (“general practice” OR “family practice”) The search was modified in an iterative manner, based on the results generated and in order to meet the search criteria of the specific data base.

### Inclusion criteria

Publications were eligible for inclusion if:

1. The publication was:

- A description of a mental health care pathway (a set of structured care methodologies, protocols, guidelines, algorithms) to guide clinical practice, or;
- A descriptive study examining trends in mental health treatment practices and pathways, practitioner perceptions and/or patient experiences, or;
- A review of one of the above.

2. The publication addressed mental health/social and emotional wellbeing:

- For young people (age range of 0–18), or;
- For Aboriginal and Torres Strait Islander Populations, or;
- In a general practice setting, or;
- All of the above criteria

3. The study was conducted in Australia
4. The abstract/full article was available in English

The inclusion criteria were intentionally designed to be broad, given that we were expecting a limited number of publications examining treatment pathways for Aboriginal young people. Broadening the inclusion criteria allowed us to make reference to evidence from the mainstream literature that may be applicable for Aboriginal young people.

### Exclusion criteria

Publications were excluded if they did not meet the above criteria. Additionally, publications were excluded if they tested a mental health intervention (randomized controlled trials, cohort studies, case control studies, pre/post), as the purpose of this study was to examine existing treatment guidelines and pathways, rather than to make recommendations about what these should contain. A large number of included studies were of a descriptive design (surveys, questionnaires, audits etc.).

## Results and discussion

Following a broad, iterative search of the literature in which 3439 articles were scanned for eligibility, we identified 70 publications that fit the inclusion criteria outlined above. Twenty-eight of these 70 publications identified specific challenges to mental health care delivery for Aboriginal young people. The majority of publications examined mental health and social and emotional wellbeing in Aboriginal young people as a general topic rather than by specific diagnoses. Two papers explicitly discussed the epidemiology, practice trends, and relevant perspectives regarding attention-deficit hyperactivity disorder in Aboriginal young people [32, 33]. One paper explicitly discussed examined anxiety and depression in this population [34]. We were unable to identify any publications that directly described trajectories by which Aboriginal young people traverse the mental health system. We were also unable to identify any evaluated pathways to mental health care among Aboriginal young people. In addition, we did not identify any published mental health care pathways to inform referral and treatment decisions for Aboriginal young people. Instead, the majority of the publications described the increased prevalence of mental health disorders among Aboriginal young people.

While we were unable to identify clearly delineated pathways to mental health care for Aboriginal young people, the included publications identified several barriers and facilitators that influence an individual’s trajectory, from help seeking to treatment. These have been summarized below.

### Barriers to the development of effective mental health care pathways for Aboriginal young people

#### Barrier 1: Lack of research into and understanding of aboriginal child and adolescent mental health

As noted above, little research to date has examined social and emotional wellbeing amongst Aboriginal children and adolescents and most publications have approached mental health broadly, with minimal examination of specific disorders. The only population-based study to date is the Western Australian Aboriginal Child Health Survey [1] and there are only two longitudinal studies that explore the health of Aboriginal children in Australia [2–4, 35]. These three large studies have resulted in multiple publications that describe various facets of mental health challenges for Aboriginal young people [1–4, 35]. Among their most salient findings, these studies report that compared to the general population of young people in Australia, Aboriginal young people experience an increased prevalence of high risk for emotional or behavioral problems and decreased contact with services [1–4, 35]. Despite this, there is little longitudinal evidence available to truly delineate the mental health trajectories of Aboriginal children, mental health service use patterns or the factors associated with improvement or progression. Further, many of the studies to date, in addition to being cross-sectional, were based on small, non-representative samples. It is thus unclear to what extent their results can be generalized and used in a nation-wide policy-making context [36].

This lack of evidence around Aboriginal child and adolescent social and emotional wellbeing is a barrier to the creation of appropriate treatment guidelines and to the delivery of culturally safe care by mainstream services in many ways. Firstly, without a validated theoretical framework, cultural competency is difficult to integrate into guidelines or teach effectively during medical training. Moreover, lack of empirical research on the topic hinders the development of evidence-informed practice or policy to drive systemic change in service delivery [14, 37]. In the absence of this necessary grounding, many mental health services are likely to struggle to provide culturally appropriate care for Aboriginal young people [37]. A lack of culturally appropriate care may in turn discourage young people and their families from accessing these services [14], contributing to poorer outcomes. This cycle may partially explain and maintain the higher prevalence of poor social and emotional wellbeing in Aboriginal communities [37].

#### Barrier 2: Limited co-ordination and collaboration between services

A study exploring the perspectives of Indigenous and non-Indigenous service providers and community members in Central Australia and the Top End of the Northern Territory found inter-service collaboration to be an essential factor in facilitating early intervention in mental health [12]. While better care coordination is seen as desirable, concern that information sharing between services or clinicians may jeopardize confidentiality was a recurring theme in the literature regarding the mental health of Aboriginal young people [10, 38, 39]. Formalized care pathways have the potential to address this concern by creating schemes in which it is clear to all parties which information should be shared, and how. Care pathways can also promote the creation of secure information systems to facilitate this process while ensuring patient confidentiality [40]. Additionally, information systems can facilitate service linkage [41], as well as information collection to inform decision making, planning, and further modification [38, 40]. Ultimately, without pathways to guide care, the roles of collaborating professionals are imprecisely defined, and there are no formalized communication routes to facilitate coordination, inter-service collaboration, information sharing, and delegation of responsibilities [10, 12, 35, 38, 40, 42, 43]. This emphasizes the need for the development of more coordinated and collaborative pathways in Aboriginal mental health, which was also echoed in two qualitative studies exploring general practice trends in Western Australia [42, 43].

### Impacts of inadequate mental health pathways for Aboriginal young people

#### Impact 1: Low help seeking

The Western Australian Aboriginal Child Health Survey [1] found that rates of mental health service use were correlated with participating Aboriginal children’s level of risk of emotional or behavioral difficulties; children at higher risk were more likely to use services than those at low risk. Overall, however, mental health service use was low. Only 8% of children aged 4–11 years who were assessed as ‘high risk’ and 22% of ‘high risk’ youth aged 12–17 years had ever had contact with mental health services, based on a review of health records [1]. Factors that may hinder help seeking include stigma [12, 24, 39, 44–46], poor accessibility of services [45, 47, 48], lack of awareness of services [39, 44, 45, 47], the perception that GPs do not provide mental health care [24, 44, 46], and reliance on informal supports instead of formal services [39, 48]. Meanwhile, caregivers who recognize mental distress among young people and have positive attitudes about help seeking can promote service use [24, 49, 50].

#### Impact 2: Under-recognition and under diagnoses

The under-recognition of mental health disorders by GPs has been identified as a primary barrier to receiving treatments and/or appropriate referrals [51]. Though we could not identify specific research examining GP confidence and accuracy in the detection of mental health problems among Aboriginal young people, the evidence suggests that GP detection rates of mental health concerns in non-Aboriginal young people are low [29, 52]. Across the literature, GP recognition has a high specificity but lower sensitivity [29, 48, 52–59]. Across multiple studies, under-recognition by GPs is concentrated among less severe cases. These cases are likely to present more subtly and to require a longer examination to identify. On the other hand, more severe cases are often recognized and responded to appropriately by GPs [18, 29, 55, 57–62]. Caregiver recognition of child and adolescent mental health problems [29, 51, 56] and self-recognition [45, 57–59] of the problem are also often related to severity, and have been found to be associated with increased service access and GP recognition.

There are multiple factors that may contribute to low rates of detection of mental health problems amongst young people by GPs. Some literature hypothesizes that, in part related to short consultation times, GPs might not be probing enough regarding the social and emotional wellbeing of their adolescent patients [46]. Adequate consultation times are critical, given that mental health concerns are often not overt, and patients may be initially reluctant to disclose [46, 63, 64]. Aboriginal mentors and Aboriginal Health Workers in urban New South Wales have emphasized the importance of allowing adequate consultation time for the relationship-building necessary for disclosure [65]. However, limited consultation time, particularly with youth, is frequently reported by GPs [24, 29, 43, 46, 50, 55, 63, 64, 66–69]. In keeping with this, feeling rushed during medical consultations has been a sentiment expressed by members of Aboriginal communities in urban New South Wales [65].

Evidence also suggests that the presentation of young people with mental disorders in primary care may be complex, with physical symptoms often presented as the main concern, complicating recognition and diagnosis [29, 59, 62, 64, 67, 70]. This pattern of presentation may also be seen in Aboriginal patients [71]; in a survey of a Central Australian Aboriginal population, Jones and de la Horne noted high rates of physical symptoms associated with mood disorders [72]. Finally, there is evidence that GPs lack confidence with respect to caring for Aboriginal young people. Specifically, children, youth, and Aboriginal populations were identified as groups that are difficult to manage by a group of rural physicians in Western Australia [43].

There is also a lack of confidence amongst GPs regarding mental health diagnosis and treatment more generally. In an Australian study, insufficient knowledge regarding proper mental health diagnosis and uncertainty regarding treatment approaches were barriers identified by 56% and 36% of GPs in the study, respectively [63]. The same study demonstrated that self-reported confidence in diagnosing and treating mental health conditions in patients was lowest for children, and varied by condition and treatment intervention [63]. Low confidence may be related to medical training; physicians trained in Australia expressed dissatisfaction with their medical training in counseling [43, 73].

It is clear that there are multiple factors that affect GP recognition of mental health disorders. Further research is required to better understand the relative impact of each factor for GPs caring for Aboriginal patients and to develop and evaluate strategies to overcoming them.

#### Impact 3: Undertreatment

There is little evidence regarding mental health treatment rates in Aboriginal populations, and thus it is difficult to conclude to what extent under-treatment is a significant problem among Aboriginal children and adolescents. However, data from the Australian Institute of Health and Welfare indicates that there are higher proportions of mental health-related presentations to the emergency room [74] and higher rates of hospitalization for mental health-related presentations [75] amongst Aboriginal people compared to non-Aboriginal people. This suggests that current treatment options for Aboriginal patients are suboptimal. Challenges to detection and treatment in an Aboriginal setting may include a lack of accessible resources, shame and stigma, and providers’ limited knowledge regarding Aboriginal culture and mental health [12].

In summary, the lack of pathways to mental health care for Aboriginal young people have multiple consequences. These include low rates of help-seeking and service use because of culturally inappropriate and poorly coordinated care. Moreover, fragmented service delivery and inadequate education and training may decrease recognition, diagnosis, and treatment rates and increase rates of patients lost to follow-up. These consequences are likely to result in worse mental health outcomes for Aboriginal young people, given the established importance of early intervention in the context of mental health distress [76–78]. This in turn is likely to have negative social, academic and health impacts for individuals, their families and communities [7, 79, 80].

### Recommendations for improved mental-health service delivery for Aboriginal young people

**Fig. 1:**
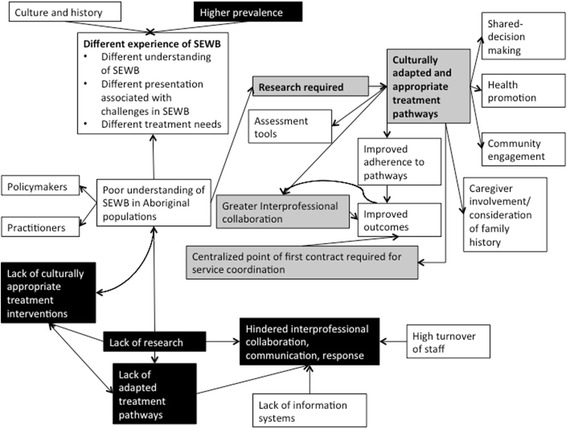
Emerging themes in the research related to the social and emotional wellbeing of Aboriginal young people. Black: Most emphasized challenges in care for Aboriginal Social and Emotional Wellbeing. Grey: Most emphasized solutions/interventions that may translate to improved services for Aboriginal young people

Given the need to develop, validate, and disseminate culturally responsive pathways, our review has identified several elements with broad support in the literature for their potential to improve Aboriginal social and emotional wellbeing (Fig. 1).

#### Facilitator 1: Central coordination and flexibility

In the development of a referral pathway model to respond to high rates of Aboriginal youth suicide and attempted suicide in Central Australia, Taylor et al. emphasized the need for a centralized point of first contact to facilitate service access and aid in the coordination of referrals. They also called for the development of data collection systems to help understand patterns and needs, to improve the targeting of responses, and to facilitate communication and feedback mechanisms within and between services [38]. These recommendations have been echoed in the literature examining mainstream Australian mental health care [40, 41]. The literature also emphasizes the need for flexibility in mental health care pathways [12, 37, 67, 81]. Specifically, pathways need to account for variations in geographic location, age, cultural background, and other demographic factors relevant to Aboriginal populations [10, 19, 42, 44, 47, 82–84].

#### Facilitator 2: Cultural modifications via caregiver and community involvement

Aboriginal Health Workers, cultural consultants, community members, elders, and consumers have the potential to inform appropriate cultural modifications. As such, these stakeholders should be actively consulted throughout the treatment process [37]. These stakeholders, as well as caregivers, family members, and support networks more broadly, act as enablers to care [10, 12, 13, 35, 38, 85]. Mohajer et al., established that family was considered to be the most important support for Aboriginal patients [85], while Williamson et al., found that Aboriginal participants felt that the observation of familial interactions and relations was fundamental to the accurate assessment of young people’s mental health [35]. Similarly, Lampe et al., in a survey of Australian GPs not limited to Aboriginal patients, reported that GPs felt that “family history [is] essential to understanding the patient and making a diagnosis” [67].

Finally, ongoing cultural awareness training and supervision for all service providers has been widely proposed, in order to ensure consistent, high quality care and promote the uptake of, and adherence to guidelines [10, 12, 37].

Our review has several strengths. Firstly, the structure of the analysis directly considers the evidence in relation to Aboriginal young people. Additionally, the conceptual mapping of themes that emerged from the literature facilitates a holistic understanding of the complex interplay of factors that affect SEWB and treatment pathways. This facilitates the uptake and application of our findings in research, policy, and practice.

The results of our review should also be considered in light of their limitations. Firstly, due to the paucity of specific, relevant evidence, a wide range of articles were used, including a wide range of methodologies, participant samples, and research questions. This limits the extent and accuracy of evidence synthesis that could be undertaken. Moreover, there was a lack of studies with representative Aboriginal populations. Similarly, the inclusion of adults (over 18 years old) in certain study samples may also limit the applicability of our analysis. It should be noted that this is not a systematic review, thus it is possible that our search did not locate all pieces of evidence that could contribute to our understanding of mental health treatment pathways for Aboriginal young people. Finally, due to the paucity of literature around specific mental health conditions amongst young people, our review focused on mental health in general and was unable to capture the nuanced differences between different disorders. When more detailed evidence becomes available, it will be important to review the evidence around specific disorders.

## Conclusions

We found that very little is known about ideal or actual pathways for the treatment of mental health concerns in Aboriginal youth. Moreover, descriptions and evaluations of current practice trends and their effectiveness are essentially non-existent. Despite this, the need for improvements in mental health service delivery is clear given the disproportionate burden of social and emotional wellbeing concerns amongst Aboriginal young people and the existing evidence, which suggests that current services are inadequate. Rigorous research is urgently needed to inform the development, implementation, and evaluation of culturally appropriate mental health care pathways for Aboriginal young people. Finally, evidence is essential to the development of sustainable policy initiatives and systemic change. Current policy priorities in Australia include decreasing Aboriginal suicide rates [86]. As such, it follows that evidence-based pathways specific to mental health problems that increase risk of suicide in Aboriginal young people are needed.

In an attempt to inform improved service delivery in Aboriginal health, we identified elements of culturally appropriate care that have wide support in the literature. These include; (1) the involvement of families and caregivers with patient/client consent; (2) an acknowledgement of the role of the community in mental health promotion, and; (3) greater interprofessional collaboration and information sharing through the establishment of central points of first contact, responsible for the coordination of service delivery and communication. Though comprehensive best practices for mental health care in this population have not yet been identified, there is significant potential for improvements in service delivery for Aboriginal young people. The input and leadership of Aboriginal Australians will be essential in trialing the implementation of the promising strategies identified here.
